# Supplementary material for: The Global Burden of Meningitis in Children: Challenges with Interpreting Global Health Estimates
Source: Microorganisms. 2021 Feb 13;9(2):377. doi: 10.3390/microorganisms9020377 (PMC7917636; doi:10.3390/microorganisms9020377)
Supplement: Supplementary file 1 [file microorganisms-09-00377-s001.pdf]

Contents

Mortality modelling methods – Meningitis, encephalitis and neonatal sepsis 1

GBD 2017 1

WHO-MCEE 2000-2017 3

Pathogen specific meningitis mortality and incidence modelling methods 4

GBD 2017 4

MCEE/JHSPH 5

Tables and Figures

Table S1: ICD10 codes mapped to meningitis, encephalitis and neonatal sepsis according to model 7

Table S2: Definitions of IHME's GBD 2017 data quality star rating 12

Table S3: Location level covariates according to model 13

Table S4: Covariates used in WHO-MCEE's multinomial logistic regression by age group, model and cause 14

Table S5: MCEE Final cause of death list 15

Table S6: MCEE modelled cause of death categories according to age 16

Figure S1: Quality of underlying cause of death data and modelling methods used to generate death estimates according to model. 17

Mortality modelling methods – Meningitis, encephalitis and neonatal sepsis

This section provides a brief overview on how the different models derive meningitis, encephalitis and neonatal sepsis mortality estimates. However, full methodology is provided by the modellers elsewhere.

Both GBD 2017 and MCEE 2000-2017 models used cause of death data from vital registration (VR) systems, sample registration systems (SR) and verbal autopsy (VA) studies to assign causes of death ensuring that the total number of deaths matches other estimates for the age-specific all-cause mortality. This involves the generation of data where data are

incomplete or completely missing. The models also try to correct for poor quality cause of death data.

A description of each model is outlined below.

GBD 2017

A core step in the GBD 2017 estimation process was the creation of a cause of death (CoD) database where cause of death data obtained from individual countries is mapped to the GBD cause list of 282 diseases and split into age and sex categories [3].

The International Classification of Diseases ICD10 codes used to map to the GBD cause categories “meningitis”, “encephalitis” and “neonatal sepsis and other neonatal infections” are outlined in Table S1.

Some causes of death were not considered specific enough to be mapped to a particular GBD cause of death category, could not be the underlying cause of death (e.g. senility) or had been assigned to the immediate or intermediate cause of death rather than the underlying cause (e.g. heart failure) and were therefore considered to be garbage codes. A statistical process was used to redistribute garbage codes to other causes of death and smooth out unrealistic data points.

For GBD 2017 meningitis death estimates, a total of 19,331 vital registration (VR) data points, 1,470 verbal autopsy (VA) data points, 793 sample registration (SR) data points and 546 surveillance data points were used [3]. A data point represents cause of death data in an individual location for a specific year. For example, VR data from a specific location for the years 1990-2017 inclusive would equate to 28 data points.

VR country-years with data less than 50% complete were dropped and country data with completeness between 50 – 69% were flagged as non-representative in the CoD database. Raw data points from VR and SR were adjusted using death distribution methods that assess the completeness of death recording relative to census recording. Raw age specific mortality was then divided by estimated completeness to account for under ascertainment in VR and SR data.

To address cause of death data that is incomplete or not available for many locations IHME used Cause of Death Ensemble Models (CODEm) to fill in the gaps in the data by drawing on data from countries with more complete data, similar characteristics and geography. Different CODEm were used to estimate meningitis deaths according to sex and in the 0 days to 4 years age group compared to 5 years and older. Additionally different CODEm models were used to estimate meningitis deaths from data rich locations (in countries with 4-star or greater rated VR systems) compared to the global model which includes all countries and data and includes those countries and territories where data was less reliable or where there was no data at all [2]. Definitions of the IHME data quality star rating is outlined in Table S2. In locations with data, the in-country data is heavily weighted, and data from the region and super region has a minor influence. In locations without data, estimates are informed by covariates and by data from the region and super region.

Location-level covariates used in CODEm models are outlined in Table S3.

Estimates generated from the CODEm models were then combined with other cause of death estimates ensuring that the sum matched the total all-cause mortality envelope for

each age group, sex, location and year. The all-cause mortality envelope is generated using a combination of surveys, censuses and vital registration data.

GBD 2017 encephalitis death estimates

Deaths from encephalitis were modelled using CODEm. The covariates used are outlined in Table S3. A total of 19,028 vital registration (VR) data points, 395 verbal autopsy (VA) data points and 793 sample registration (SR) data points were used [3].

GBD 2017 neonatal sepsis death estimates

Deaths from neonatal sepsis were modelled in children under 5 years using CODEm in four separate age groups: Early neonatal period, late neonatal period, post neonatal period and 1-4 years. The covariates used are outlined in Table S3. A total of 18,175 vital registration (VR) data points, 165 verbal autopsy (VA) data points and 791 sample registration (SR) data points were used [3]. Modellers excluded the majority of verbal autopsy data (apart from in India) in the estimation of neonatal sepsis deaths because validation studies indicate that verbal autopsy methods are less accurate or defining cause of death in this age group.

WHO-MCEE 2000-2017

WHO-MCEE calculated cause of death fractions according to a predefined cause list (Table S5). These cause of death fractions were then applied to neonatal and 1-59 month all-cause estimates produced by UN-IGME. The underlying data used by UN-IGME for calculating mortality rate and deaths comes from surveys, censuses and vital registration data.

Three methods were used to calculate cause of death fractions for a country depending on the quality of the cause of death data available for that country and its mortality setting.

VR data - For countries with high quality data covering >80% of the population VR data was used directly to estimate cause of death fractions attributed to the cause categories described in Table S5. Data was defined as high quality if countries had reported at least five years of data to WHO with an average usability over this period of equal to or over 80%. Usability is calculated as completeness of data multiplied by the proportion of registered deaths that are assigned a meaningful cause [27, 49]. The ICD10 codes used to map to meningitis, encephalitis and neonatal sepsis are provided in Table S1.

VRMCM – In low mortality countries (<35 deaths/1000 live births 2000-2010) data from the countries with high quality VR were used to fit a multinomial logistic regression model, called the vital registration multi-cause model (VRMCM), using the covariates outlined in Tables 3 and 4. Cause of death fractions were attributed to the cause categories described in Table S6.

VAMCM - In high mortality countries (>35 deaths/1000 live births 2000-2010) the cause distribution was estimated using a multinomial model applied to verbal autopsy data. The verbal autopsy multi-cause model (VAMCM) was a multinomial logistic regression model fitted using VA data from 119 studies in 39 countries using the covariates outlined in Tables 3 and 4. Cause of death fractions were attributed to the cause categories described in Table S6.

Causes of death within the early neonatal (0-6 days), late neonatal (7-28 days) and post neonatal period (0-11 months) were modelled separately from each other because the cause of death distributions can differ significantly within these age groups. Different country specific covariates were used to derive the predictions based on age, model type and cause

(Table S4). Despite the early and late neonatal period being modelled separately, only neonatal and post neonatal cause of death estimates are published. Post –hoc adjustment covariates were also used to account for meningitis deaths averted by PCV and Hib vaccines. The adjustments take into account serotype coverage of the vaccine in the case of PCV, vaccine coverage and vaccine effectiveness.

In the MCEE 2000-2017 estimation round neonatal meningitis was estimated separately from neonatal sepsis for the first time. These causes were estimated separately for the first time in their latest modelling round by using the ratio of neonatal meningitis and neonatal sepsis deaths derived from IHME estimates. The six recognised direct causes of neonatal deaths identifiable by verbal autopsy are: (1) serious infection (including sepsis, pneumonia and meningitis), (2) birth asphyxia, (3) prematurity, (4) tetanus, (5) congenital malformation and (6) diarrhoea [50].

Pathogen specific meningitis mortality and incidence modelling methods

GBD 2017

Pathogen specific mortality estimates

GBD 2017 assigned overall deaths from meningitis as predicted using CODEm into pathogenic causes using a set of proportional models in DisMod-MR 2.1. Proportions were informed using vital registration death data coded down to cause-level. The meningococcal meningitis proportion model used two country level covariates (the proportion of the population living in the meningitis belt and the proportion of the population covered by the MenAfriVac vaccine). The pneumococcal meningitis proportion model used PCV3 vaccine coverage as a covariate and the Hib model used Hib3 vaccine coverage as a covariate. The other meningitis proportion model included Hib3 vaccine coverage, pneumococcal vaccine coverage, and the proportion of people living in the meningitis belt. The four proportion models were scaled to sum to 100% for each location, age-group, sex, and year combination to convert meningitis deaths into meningitis deaths by aetiology [3].

CODEm smooths VR or VA data over time which can result in spikes caused by outbreaks not being represented in the data. For this reason meningococcal meningitis outbreaks were estimated as a fatal discontinuity which means they were added to overall meningitis deaths after they were corrected to fit within remainder of all-cause mortality envelope (CODCorrect). The Global Infectious Disease and Epidemiology Network (GIDEON) and WHO death reports were used as the data sources for epidemic meningococcal meningitis deaths.

Pathogen specific incidence estimates

Overall incidence of acute bacterial meningitis was modelled using DisMod- MR 2.1 informed by incidence data gathered from hospital records, claims data and a systematic review of the literature capturing incidence studies. In total 5,535 site-years of incidence data fed into the model [51]. DisMod- MR 2.1 pools all the available incidence data adjusting for systematic bias associated with the source of the data and its variance from a reference data source which in this case was ICD coded hospital data. Three country level covariates (proportion of the population living in the meningitis belt, coverage of Hib3 vaccine and coverage of MenAfriVac vaccine) were also applied in locations where data was lacking.

Meningitis incidence was then assigned by aetiology using a second set of DisMod- MR 2.1 proportion models. Proportions by aetiology were informed by surveillance data and

literature that reports cause fractions. A Hib vaccine coverage covariate was applied to the Hib proportion model, the proportion of the population living in the meningitis belt and coverage of MenAfriVac vaccine was applied to the meningococcal meningitis proportion model and PCV3 coverage covariate applied to the pneumococcal meningitis model. Data sources encompassed both epidemic and non-epidemic years, but cases arising as a result of meningococcal meningitis outbreaks were not added to incidence estimates separately.

MCEE/JHSPH

Pathogen specific mortality estimates

The MCEE/JHSPH pathogenic model assigned pathogenic causes to estimated deaths from meningitis/encephalitis from the WHO/MCEE 2000-2015 syndromic model, adjusted to represent no vaccine use. For the purposes of the model the entire meningitis/encephalitis envelope was assumed to be meningitis.

Proportions of deaths due to each pathogen were informed by a meta-analysis of meningitis case aetiology distribution pre-vaccine era (stratified by region). It was assumed that 88% of meningitis in the pre-vaccine era was caused by pneumococcal, Hib and meningococcal bacteria combined. As the literature on case aetiology distribution was relatively rich and aetiology of mortality distribution relatively poor, poor case aetiology distribution was converted into proportions of deaths using relative pathogen specific case fatality rates (stratified by child mortality setting). Once country and pathogen- specific meningitis deaths prior to vaccine use had been calculated, adjustments were made to account for country specific Hib and pneumococcal vaccine coverage [14].

WHO/MCEE account for children infected with human immunodeficiency virus (HIV) who die from meningitis in HIV/AIDS death estimates [52]. MCEE/JHSPH include deaths from meningitis which occurred in HIV-positive children by applying relative risks for invasive pneumococcal and Hib disease to annual estimates of HIV prevalence [14].

Pathogen specific incidence estimates

To calculate meningitis incidence according to pathogen, pathogen-specific deaths according to country were divided by country and pathogen specific meningitis case fatality ratio estimates. In areas with low health seeking behaviour for pneumonia symptoms, as derived from Demographic and Health Surveys and UNICEF's Multiple Indicator Cluster Surveys, a case fatality rate of 90% was assumed.

Table S1: ICD10 codes mapped to meningitis, encephalitis and neonatal sepsis according to model

	IHME	MCEE
	Meningitis/	
	Encephalitis	Meningitis

A39.0 Meningococcal meningitis

A39.1 - Waterhouse-Friderichsen syndrome (Meningococcal haemorrhagic adrenalitis, Meningococcic adrenal syndrome)

A39.2 - Acute meningococcaemia

A39.3 - Chronic meningococcaemia

A39.4 - Meningococcaemia

A39.8 - Other meningococcal infections

A39.9 - Meningococcal infection, unspecified

A87.0 - Enteroviral meningitis (G02.0*)

A87.1 - Adenoviral meningitis (G02.0*)

A87.2 - Lymphocytic choriomeningitis

A87.8 - Other viral meningitis

A87.9 - Viral meningitis, unspecified

D86.81 - [D86.8] Sarcoidosis of other and combined site

G00.0 - Haemophilus meningitis

G00.1 - Pneumococcal meningitis

G00.9 – Bacterial meningitis unspecified

G01 – Meningitis in bacterial diseases classified elsewhere

G02 - Meningitis in other infectious and parasitic diseases classified elsewhere

G02.0 – Meningitis in viral diseases classified elsewhere

G02.1 - Meningitis in mycoses

G02.8 – Meningitis in other specified infectious and parasitic diseases classified elsewhere

G03.9 – Meningitis unspecified

G03.1 - Chronic meningitis

G03.2 - Benign recurrent meningitis (Mollaret)

G03.8 - Meningitis due to other specified causes

Encephalitis

A83.0 - Japanese encephalitis

A83.1 - Western equine encephalitis

A83.2 - Eastern equine encephalitis

A83.3 - St Louis encephalitis

A83.4 - Australian encephalitis

A83.5 - California encephalitis

A83.6 - Rocio virus disease

A83.8 - Other mosquito-borne viral encephalitis

A83.9 - Mosquito-borne viral encephalitis, unspecified

A84.0 Far Eastern tick-borne encephalitis [Russian spring-summer encephalitis]

A84.1 central European Tick-borne encephalitis

A84.8 Other tick-borne viral encephalitis

A84.9 Tick-borne viral encephalitis, unspecified

A85.0 Enteroviral encephalitis (G05.1*)

Enteroviral encephalomyelitis

A85.1 Adenoviral encephalitis (G05.1*)

Adenoviral meningoencephalitis

A85.2 Arthropod-borne viral encephalitis, unspecified

A85.8 Other specified viral encephalitis

A86 Unspecified viral encephalitis

B94.1 Sequelae of viral encephalitis

F07.1 Post encephalitic syndrome

G04.0 - Acute disseminated encephalitis

G04.1 - Tropical spastic paraplegia

G04.2 - Bacterial meningoencephalitis and meningomyelitis, not elsewhere classifed

G04.8 - Other encephalitis, myelitis and encephalomyelitis

G04.9 - Encephalitis, myelitis and encephalomyelitis, unspecified

G05.0* - Encephalitis, myelitis and encephalomyelitis in bacterial diseases classified elsewhere

G05.1* - Encephalitis, myelitis and encephalomyelitis in viral diseases classified elsewhere

G05.2* - Encephalitis, myelitis and encephalomyelitis in other infectious and parasitic diseases classified elsewhere

G05.8* - Encephalitis, myelitis and encephalomyelitis in other diseases classified elsewhere

G21.3 Postencephalitic parkinsonism A20.3 - Plague meningitis

A32.1 - Listerial meningitis and meningoencephalitis

A39.0 - Meningococcal meningitis (G01*)

A39.1 - Waterhouse-Friderichsen syndrome (Meningococcal haemorrhagic adrenalitis, Meningococcic adrenal syndrome)

A39.2 - Acute meningococcaemia

A39.3 - Chronic meningococcaemia

A39.4 - Meningococcaemia

A39.5 - Meningococcal heart disease

A39.8 - Other meningococcal infections

A39.9 - Meningococcal infection, unspecified

A83.0 - Japanese encephalitis

A83.1 - Western equine encephalitis

A83.2 - Eastern equine encephalitis

A83.3 - St Louis encephalitis

A83.4 - Australian encephalitis

A83.5 - California encephalitis

A83.6 - Rocio virus disease

A83.8 - Other mosquito-borne viral encephalitis

A83.9 - Mosquito-borne viral encephalitis, unspecified

A87.0 - Enteroviral meningitis (G02.0*)

A87.1 - Adenoviral meningitis (G02.0*)

A87.2 - Lymphocytic choriomeningitis

A87.8 - Other viral meningitis

A87.9 - Viral meningitis, unspecified

G00.0 - Haemophilus meningitis

G00.1 - Pneumococcal meningitis

G00.2 - Streptococcal meningitis

G00.3 - Staphylococcal meningitis

G00.8 - Other bacterial meningitis (E.coli, Friedlander bacillus, Klebsiella)

G00.9 - Bacterial meningitis, unspecified (purulent NOS, pyogenic NOS, suppurative NOS)

G03.0 - Nonpyogenic meningitis

G03.1 - Chronic meningitis

G03.2 - Benign recurrent meningitis (Mollaret)

G03.8 - Meningitis due to other specified causes

G03.9 - Meningitis, unspecified

G04.0 - Acute disseminated encephalitis

G04.1 - Tropical spastic paraplegia

G04.2 - Bacterial meningoencephalitis and meningomyelitis, not elsewhere classifed

G04.8 - Other encephalitis, myelitis and encephalomyelitis

G04.9 - Encephalitis, myelitis and encephalomyelitis, unspecified

Neonatal Sepsis P36.0 - Sepsis of newborn due to streptococcus, group B

P36.1 - Sepsis of newborn due to other and unspecified streptococci

P36.2 - Sepsis of newborn due to staphylococcus aureus

P36.3 - Sepsis of newborn due to other and unspecified staphylococci

P36.4 - Sepsis of newborn due to Escherichia coli

P36.5 - Sepsis of newborn due to anaerobes

P36.8 - Other bacterial sepsis of newborn

P36.9 - Bacterial sepsis of newborn, unspecified

P38 - Omphalitis of newborn with or without mild haemorrhage

P39.9 - Infection specific to the perinatal period, unspecified A15 - Respiratory tuberculosis, bacteriologically and histologically confirmed

A20.2 Pneumonic plague

A20.7 - Septicaemic plague

A20.8 - Other forms of plague

A20.9 - Plague, unspecified

A21 - Tularaemia

A22 - Anthrax

A23 - Brucellosis

A24 - Glanders and melioidosis

A25 - Rat-bite fevers

A26 - Erysipeloid

A27 - Leptospirosis

A28 - Other zoonotic bacterial diseases, not elsewhere classified

A30 - Leprosy

A31 - Infection due to other mycobacteria

A32.0 - Cutaneous listeriosis

A32.7 - Listerial sepsis

A32.9 - Listeriosis, unspecified

A38 – Scarlet fever

A40 - Streptococcal sepsis

A41 - Other sepsis

A42 - Actinomycosis

A43 - Nocardiosis

A44 - Bartonellosis

A46 - Erysipelas

A48 - Other bacterial diseases, not elsewhere classified

A49 - Bacterial infection of unspecified site

A50 - Congenital syphilis

A51 - Early syphilis

A52 - Late syphilis

A53 - Other and unspecified syphilis

A54 - Gonococcal infection

A55 - Chlamydial lymphogranuloma (venereum)

A56 - Other sexually transmitted chlamydial diseases

A57 - Chancroid

A58 - Granuloma inguinale

A59 - Trichomoniasis

A60 - Anogenital herpesviral [herpes simplex] infection

A63 - Other predominantly sexually transmitted diseases, not elsewhere classified

A64 - Unspecified sexually transmitted disease

A65 - Nonvenereal syphilis

A66 - Yaws

A67 - Pinta [carate]

A68 - Relapsing fevers

A69 - Other spirochaetal infections

A70 - Chlamydia psittaci infection

A71 - Trachoma

A74 - Other diseases caused by chlamydiae

A75 - Typhus fever

A77 - Spotted fever [tick-borne rickettsioses]

A78 - Q fever

A79 - Other rickettsioses

A80 - Acute poliomyelitis

A81 - Atypical virus infections of central nervous system

A82 - Rabies

A88 - Other viral infections of central nervous system, not elsewhere classified

A89 - Unspecified viral infection of central nervous system

A90 - Dengue fever [classical dengue]

A91 - Dengue haemorrhagic fever

A92 - Other mosquito-borne viral fevers

A93 - Other arthropod-borne viral fevers, not elsewhere classified

A94 - Unspecified arthropod-borne viral fever

A95 - Yellow fever

A96 - Arenaviral haemorrhagic fever

A98 - Other viral haemorrhagic fevers, not elsewhere classified

A99 - Unspecified viral haemorrhagic fever

B00 - Herpesviral [herpes simplex] infections

B01 - Varicella [chickenpox]

B02 - Zoster [herpes zoster]

B03 – Smallpox

B04 – Monkeypox

B05 – Measles

B06 - Rubella [German measles]

B07 - Viral warts

B08 - Other viral infections characterized by skin and mucous membrane lesions, not elsewhere classified

B09 - Unspecified viral infection characterized by skin and mucous membrane lesions

B15 - Acute hepatitis A

B16 - Acute hepatitis B

B17 - Other acute viral hepatitis

B18 - Chronic viral hepatitis

B19 - Unspecified viral hepatitis

B25 - Cytomegaloviral disease

B26 – Mumps

B27 - Infectious mononucleosis

B30 - Viral conjunctivitis

B33 - Other viral diseases, not elsewhere classified

B34 - Viral infection of unspecified site

B35 – Dermatophytosis

B36 - Other superficial mycoses

B37 – Candidiasis

B38 – Coccidioidomycosis

B39 – Histoplasmosis

B40 – Blastomycosis

B41 – Paracoccidioidomycosis

B42 – Sporotrichosis

B43 - Chromomycosis and phaeomycotic abscess

B44 – Aspergillosis

B45 – Cryptococcosis

B46 – Zygomycosis

B47 – Mycetoma

B48 - Other mycoses, not elsewhere classified

B49 - Unspecified mycosis

B55 – Leishmaniasis

B56 - African trypanosomiasis

B57 - Chagas disease

B58 – Toxoplasmosis

B59 – Pneumocystosis

B60 - Other protozoal diseases, not elsewhere classified

B64 - Unspecified protozoal disease

B65 – B83 Helminthiases

B85 – B89 - Pediculosis, acariasis and other infestations

B90 – B94 - Sequelae of infectious and parasitic diseases

B95 - Streptococcus and staphylococcus as the cause of diseases classified to other chapters

B96 - Other specified bacterial agents as the cause of diseases classified to other chapters

B97 - Viral agents as the cause of diseases classified to other chapters

B98 - Other specified infectious agents as the cause of diseases classified to other chapters

B99 - Other and unspecified infectious diseases

G01 - Meningitis in bacterial diseases classified elsewhere

G02 - Meningitis in other infectious and parasitic diseases classified elsewhere

G05.0 - Encephalitis, myelitis and encephalomyelitis in bacterial diseases classified elsewhere

G05.1 - Encephalitis, myelitis and encephalomyelitis in viral diseases classified elsewhere

G05.2 - Encephalitis, myelitis and encephalomyelitis in other infectious and parasitic disease classified elsewhere

G05.8 - Encephalitis, myelitis and encephalotmyelitis in other diseases classified elsewhere

G06.0 - Intracranial abscess and granuloma

G06.1 - Intraspinal abscess and granuloma

G06.2 - Extradural and subdural abscess, unspecified

G07 - Intracranial and intraspinal abscess and granuloma in disease classified elsewhere

G08 - Intracranial and intraspinal phlebitis and thrombophlebitis

G09 - Sequelae of inflammatory diseases of central nervous system

P35.0 - Congenital rubella syndrome

P35.1 - Congenital cytomegalovirus infection

P35.2 - Congenital herpesviral [herpes simplex] infection

P35.3 - Congenital viral hepatitis

P35.8 - Other congenital viral diseases

P35.9 - Congenital viral disease, unspecified

P36.0 - Sepsis of newborn due to streptococcus, group B

P36.1 - Sepsis of newborn due to other and unspecified streptococci

P36.2 - Sepsis of newborn due to staphylococcus aureus

P36.3 - Sepsis of newborn due to other and unspecified staphylococci

P36.4 - Sepsis of newborn due to Escherichia coli

P36.5 - Sepsis of newborn due to anaerobes

P36.8 - Other bacterial sepsis of newborn

P36.9 - Bacterial sepsis of newborn, unspecified

P37.0 - Congenital tuberculosis

P37.1 - Congenital toxoplasmosis

P37.2 - Neonatal (disseminated) listeriosis

P37.5 - Neonatal candidiasis

P37.8 - Other specified congenital infectious and parasitic diseases

P37.9 - Congenital infectious and parasitic disease, unspecified

P38 - Omphalitis of newborn with or without mild haemorrhage

P39.0 - Neonatal infective mastitis

P39.1 - Neonatal conjunctivitis and dacryocystitis

P39.2 - Intra-amniotic infection of fetus, not eslsewhere classified

P39.3 - Neonatal urinary tract infection

P39.4 - Neonatal skin infection

P39.8 - Other specified infections specific to the perinatal period

P39.9 - Infection specific to the perinatal period, unspecified

Table S2: Definitions of IHME's GBD 2017 data quality star rating

Data quality star rating	Definition
5 stars	85%-100% well-certified
4 stars	65%-84% well-certified
3 stars	35%-64% well-certified
2 stars	10%-34% well-certified
1 star	>0%-9% well-certified
0 stars	No vital registration or verbal autopsy data available from 1980-2017

% well-certified is a function of the completeness of the cause of death data multiplied by the quality of the data – e.g. the proportion of deaths registered to a well-defined cause.

Table S3: Location level covariates according to model

GBD 2017 location level covariates used in CODEm according to cause of death Full list of potential WHO-MCEE location level covariates used in VRMCM and/or VAMCM

Included in all models (meningitis/encephalitis and neonatal sepsis) Proportion of children <5 yrs who are underweight

Healthcare access and Quality Index

Health system access (composite of vaccine coverage and pregnancy services),

Lag distributed income per capita (I$),

Maternal education (years per capita) Female literacy

Gini coefficient

Neonatal mortality rate

Infant mortality rate

Under 5 mortality rate

Under 5 population size

Low birth weight

GNI per capita (PPP, $international)

Human development index

Education index

Antenatal care coverage

Percentage of births with skilled birth attendance

Percent urbanisation

Percent with access to improved drinking water

General fertility rate

Neonates protected at birth against neonatal tetanus

Percent low birth weight

Plasmodium falciparum parasite rate

Meningitis epidemic

BCG vaccine coverage

PAB vaccine coverage

DTP3 vaccine coverage

Hib3 vaccine coverage

Measles vaccine coverage

Year

WHO region

Meningitis Proportion of population living in the meningitis belt

Proportion of households with access to improved water

DTP3 vaccine coverage

Hib3 vaccine coverage

MenAfriVac vaccine coverage from 2010 to 2012

Sociodemographic index,

Sanitation (proportion with access)

Encephalitis Japanese encephalitis binary,

DTP3 coverage,

Proportion of in-facility deliveries,

Sanitation (proportion with access),

Water (proportion with access),

Socio-demographic Index

Neonatal sepsis Indoor air pollution (all cooking fuels),

Smoking prevalence (reproductive age-standardized),

Antenatal care (4 visits) coverage (proportion),

In-facility delivery (proportion),

Live births 35+ (proportion),

Skilled birth attendance (proportion),

Age-standardised underweight (weight-for-age) SEV,

Total fertility rate,

Socio-demographic Index

Table S4: Covariates used in WHO-MCEE’s multinomial logistic regression by age group, model and cause

Cause Age group VRMCM VAMCM

Meningitis 1-59 months Region

Year

Human development index

Hib3 vaccine coverage GNI per capita (PPP, $international)

Measles vaccine coverage

Meningitis epidemic

Sepsis and other severe infections Early neonatal Gini coefficient

Infant mortality rate

BCG vaccine coverage

Period

Low birth weight

Late neonatal Gini coefficient

Low birth weight

Neonatal mortality rate Antenatal care coverage

Female literacy

Period

Neonates protected at birth against neonatal tetanus

Perinatal (inc. sepsis) 1-59 months Region,

Hib3 vaccine coverage,DTP3 vaccine coverage

Under 5 mortality rate

Percentage of births with skilled birth attendance

Percent with access to improved drinking water Period,

Underweight

Table S5: MCEE Final cause of death list

HIV/AIDS

Complications of preterm birth

Intrapartum-related complications

Congenital anomalies

Pneumonia

Diarrhoea

Tetanus

Meningitis/encephalitis

Sepsis and other infectious conditions of the newborn*

Malaria**

Measles**

Injuries

Other communicable diseases

Other non-communicable diseases

*Neonatal only

**Postneonatal only

Table S6: MCEE modelled cause of death categories according to age

Neonatal cause of death categories	Post neonatal cause of death categories
Complications of preterm birth	Pneumonia
Intrapartum-related complications	Diarrhoea
Congenital disorders	Malaria*
Pneumonia	Meningitis
Diarrhoea	Injuries
Neonatal tetanus*	Congenital malformations
Sepsis and other severe infections	Perinatal
Injuries	Other non-communicable diseases**
Other causes	Other causes

*VAMCM only – not modelled in VRMCM

**VRMCM only – not modelled in VAMCM

Figure S1: Quality of underlying cause of death data and modelling methods used to generate death estimates according to model.

References

Refer to main paper "The Global Burden of Meningitis in Children: Challenges with Interpreting Global Health Estimates"
